# A continuum of genetic liability for minor and major depression

**DOI:** 10.1038/tp.2017.99

**Published:** 2017-05-16

**Authors:** E C Corfield, Y Yang, N G Martin, D R Nyholt

**Affiliations:** 1 Faculty of Health, Institute of Health and Biomedical Innovation, Queensland University of Technology, Brisbane, QLD, Australia; 2 QIMR Berghofer Medical Research Institute, Brisbane, QLD, Australia

## Abstract

The recent success of a large genome-wide association (GWA) study—analysing 130 620 major depression cases and 347 620 controls—in identifying the first single-nucleotide polymorphism (SNP) loci robustly associated with major depression in Europeans confirms that immense sample sizes are required to identify risk loci for depression. Given the phenotypic similarity between major depressive disorder (MDD) and the less severe minor depressive disorder (MiDD), we hypothesised that broadening the case definition to include MiDD may be an efficient approach to increase sample sizes in GWA studies of depression. By analysing two large twin pair cohorts, we show that minor depression and major depression lie on a single genetic continuum, with major depression being more severe but not aetiologically distinct from minor depression. Furthermore, we estimate heritabilities of 37% for minor depression, 46% for major depression and 48% for minor or major depression in a cohort of older adults (aged 50–92). However, the heritability of minor or major depression was estimated at 40% in a cohort of younger adults (aged 23–38). Moreover, two robust major depression-risk SNPs nominally associated with major depression in our Australian GWA data set produced more significant evidence for association with minor or major depression. Hence, broadening the case phenotype in GWA studies to include subthreshold definitions, such as MiDD, should facilitate the identification of additional genetic risk loci for depression.

## Introduction

Major depressive disorder (MDD) is a common, complex trait with an estimated genetic heritability of ~40%.^1^ However, until recently, genome-wide association (GWA) studies in large European samples have failed to robustly identify genetic variants contributing to MDD.^2, 3, 4, 5, 6, 7, 8, 9, 10, 11^ In July 2015, two genome-wide significant (*P*<5 × 10^−8^) single-nucleotide polymorphism (SNP) loci (rs12415800 near the *SIRT1* gene, *P*=2.53 × 10^−10^, and rs35936514 in the intron of *LHPP*, *P*=6.45 × 10^−12^) were reported to be associated with severe and recurrent MDD in a sample of Han Chinese women (5282 cases and 5220 controls);^12^ however, these SNPs were not associated in the Psychiatric Genomics Consortium GWA study of 9240 European MDD cases and 9519 controls.^11^

In August 2016, the first SNP loci robustly associated with major depression in Europeans were reported.^13^ This landmark study analysed a combined cohort of 130 620 self-reported and clinically evaluated lifetime major depression cases and 347 620 controls, and identified 17 genome-wide significant SNPs within 15 independent genomic regions. The implicated SNP risk loci comprise rs10514299 in an intron of *TMEM161B-AS1* (*P*=9.99 × 10^−16^), rs1518395 in an intron of *VRK2* (*P*=4.32 × 10^−12^), rs2179744 in an intron of *L3MBTL2* (*P*=6.03 × 10^−11^), rs11209948 downstream of *NEGR1* (*P*=8.38 × 10^−11^), rs454214 upstream of *MEF2C* (*P*=1.09 × 10^−9^), rs301806 in an intron of *RERE* (*P*=1.90 × 10^−9^), rs1475120 in an intron of *LIN28B* (*P*=4.17 × 10^−9^), rs10786831 in an intron of *SORCS3* (*P*=8.11 × 10^−9^), rs12552 in the 3′ untranslated repeat of *OLFM4* (*P*=8.16 × 10^−9^), rs6476606 in an intron of *PAX5* (*P*=1.20 × 10^−8^), rs8025231 in an intergenic region between *MEIS2* and *TMCO5A* (*P*=1.23 × 10^−8^), rs12065553 in an intergenic region on chromosome 1 (*P*=1.32 × 10^−8^), rs1656369 in the intergenic region between *RSRC1* and *MLF1* (*P*=1.34 × 10^−8^), rs4543289 in an intergenic region on chromosome 5 (*P*=1.36 × 10^−8^), rs2125716 upstream of *SLC6A15* (*P*=3.05 × 10^−8^), rs2422321 downstream of *NEGR1* (*P*=3.18 × 10^−8^) and rs7044150 in the intergenic region between *KIAA0020* and *RFX3* (*P*=4.31 × 10^−8^). An important implication of this study is that immense sample sizes are required to identify a relatively modest number of MDD risk loci in Europeans (compared to other traits of similar prevalence and heritability).^14^

Additional insights into the molecular mechanisms of depression, in Europeans, were identified in 2016 through the investigation of depressive symptoms and a broad depression phenotype. In April, rs7973260 in an intron of *KSR2* (*P*=1.8 × 10^−9^) and rs62100776 in an intron of *DCC* (*P*=8.5 × 10^−9^) were associated with depressive symptoms.^15^ Meanwhile, in December, rs9825823 located in the intron of *FHIT* (*P*=8.2 × 10^−9^) was associated with a broad depression phenotype—including MDD and depressive symptoms.^16^ Most recently, MDD in adults aged over 27 years was associated with the intergenic SNP rs7647854 located on chromosome 3 (*P*=5.2 × 10^−11^).^17^

Given the phenotypic similarity between MDD and the less severe minor depressive disorder (MiDD), we hypothesised that broadening the case definition to include subthreshold definitions such as MiDD may provide an efficient means to increase sample sizes in GWA studies of depression.

In contrast to MDD, the heritability and genetics of MiDD have not been well investigated. The only difference in diagnosis between MDD and MiDD is the number of presenting symptoms of the Diagnostic and Statistical Manual of Mental Disorders (DSM-IV) criteria (NB, within the DSM-V, MiDD individuals would be diagnosed as ‘Unspecified Depressive Disorder’), with MDD requiring at least five symptoms and MiDD requiring two to four symptoms.^18, 19^ This phenotypic similarity coupled with a reported increased risk of MDD in first-degree relatives and patients with MiDD^20, 21, 22, 23, 24, 25^ suggests that a depression continuum exists, and that MiDD may be a relevant trait which could be utilised to elucidate the underlying mechanisms associated with MDD. Therefore, the present study utilises relative risks (RR) to ensure a similar pattern of familiarity exists within the study cohorts. In addition, heritability estimates and the liability threshold model were utilised to investigate whether minor and major depression lie on a single genetic continuum. Finally, the association signal of the 17 SNPs robustly associated with major depression in Europeans was examined utilising both a narrow major depression case phenotype and a broader depression phenotype including minor depression (that is, minor or major depression cases).

## Materials and methods

### Study cohorts

Two independent, community-based cohorts of Australian twin pairs were analysed within the current study. Initially, the analysis was conducted within an older adult cohort, the over 50's (aged) study, before being replicated in a young adult cohort, the Twin 89 (TE) study. Informed written consent was obtained from each participant, and the studies were approved by the Human Research Ethics Committee of the QIMR Berghofer Medical Research Institute.

The over 50’s cohort^26, 27^ contained 1220 twin pairs with complete self-reported depression classifications (non-depressed, minor depression, major depression). Current depression classifications were obtained utilising a combination of responses from the 12-item General Health Questionnaire (GHQ)^28^ and the 14-item Delusions-Symptoms-States Inventory, States of Anxiety and Depression (DSSI/sAD)^29^ questionnaires. As previously detailed,^30^ specific questions from the GHQ and DSSI/sAD were assigned to the appropriate DSM-IV major depressive episode criteria.^19^ If an individual exhibited at least five of the DSM-IV symptom criteria, of which either depressed mood or anhedonia was reported, they were assessed as suffering major depression. Similarly, if an individual exhibited two to four of the DSM-IV symptom criteria (depressed mood, anhedonia, a change in weight or appetite, insomnia or hypersomnia, psychomotor agitation or retardation, fatigue or loss of energy, feelings of worthlessness or excessive guilt, inability to concentrate or make decisions and thoughts about death, suicidal thoughts, suicidal plans or suicidal attempts), of which either depressed mood or anhedonia was reported, they were assessed as suffering minor depression. The remaining individuals were assessed as non-depressed. Meanwhile, the TE cohort^31^ contained 2363 twin pairs, with complete lifetime self-reported depression classifications. Minor depression and major depression classifications required an individual to report depressed mood and/or anhedonia. In addition, individuals reporting a total of two to four and five or more depression symptoms for a period of 2 or more weeks, across their lifespan, were classified as minor depression and major depression, respectively. In-depth explanation of depression assessment utilised within the TE cohort is provided by Yang *et al.*^32^

### Statistical analysis

Familial clustering of major depression, minor depression and depression (minor or major depression) was investigated by calculating RR with their 95% confidence intervals (CI) in monozygotic (MZ) and dizygotic (DZ) twin pairs. RRs were calculated relative to non-depressed individuals. Within MZ and same-sex DZ twin pairs, RRs were calculated by averaging over using twin 1 or twin 2 as the proband.^33^

A major goal of the genetic analysis was to test the multiple-threshold model, which asserts that different syndromes reflect different levels of severity on a single dimension, rather than distinct aetiologies.^34^ The fit of the multiple-threshold model was tested by calculating the polychoric correlation for the three-category depression (non-depressed, minor depression and major depression) classification using POLYCORR.^35^ The polychoric correlation assumes that, underlying the observed polychotomous distribution of affection status, there exists a continuous, normally distributed latent (non-observable) liability.^36^ That is, the polychoric correlation is an estimate of the correlation between two latent variables, where each latent variable is assumed to have a bivariate normal distribution. A *χ*^2^-goodness-of-fit test is used to test whether the multiple-threshold model provides a good fit to the observed data (that is, compares the observed frequencies to those predicted by the model). In addition, polychoric correlations were calculated for minor depression (excluding major depression cases), major depression (excluding minor depression cases), two-category depression (non-depressed, minor or major depression) and three-category depression within MZ and DZ twin pairs. Comparisons of the correlations between MZ and DZ twin pairs were used to provide information on the importance of genetic and environmental factors contribution to the heritability of depression.

Structural equation modelling was utilised to investigate the heritability of minor depression (excluding major depression cases), major depression (excluding minor depression cases), two-category depression and three-category depression. Structural equation modelling was used to estimate the contribution of additive genetic (A), non-additive (dominance) genetic (D), common environmental (C) and unique environmental (E) variance components.^37^ Adjustments for (linear) age and sex effects were included in the model. Significance of the variance components was assessed by comparing the fit of the full model (ACE/ADE) to the nested models (AE, CE and E) where the effect was dropped, using OpenMx in R.^38^ The goodness-of-fit parameters used to assess the differences in the twin models were the likelihood-ratio *χ*^2^-test and the *P*-value. In addition, model fit was compared utilising Akaike’s Information Criteria (AIC), with the lowest AIC indicating the most parsimonious model.^39, 40^

An association analysis was conducted for the 17 genome-wide significant loci associated with major depression, in Europeans,^13^ within the Australian GWA data set. The Australian GWA data set is a community cohort that contained 3664 unrelated major depression cases, 620 unrelated minor depression cases and 7113 unrelated controls of European ancestry. In-depth explanation of the genotyping and quality-control methods utilised within the GWA cohort has previously been detailed by Medland *et al.*^41^ Briefly, standard quality-control measures were utilised, whereby SNPs with BeadStudio GenCall scores <0.7, call rate<0.95, Hardy–Weinberg equilibrium *P*-values<1 × 10^−6^ and minor allele frequencies <0.01 were excluded. Imputation was then conducted utilising HapMap samples of European ancestry. If multiple cases were present within a family, the most severe case was selected based on the number of reported DSM depression symptoms, or an individual was randomly selected if numerous individuals reported the same number of depression symptoms. Similarly, if multiple controls were available within a family, the single ‘best’ control was selected based on the lowest number of depression symptoms reported or an individual was randomly selected if numerous individuals within a family reported the same number of depression symptoms. Finally, a single population control was randomly selected from the remaining families for whom genotyping data but no depression phenotype data were available.

The association analysis was conducted on 3664 major depression cases and 7113 controls, using logistic regression with sex as a covariate, using PLINK.^42^ The association analysis was then repeated using a broad depression phenotype, of 4284 cases (3664 major depression+620 minor depression cases). The results were then compared to ascertain whether the evidence for association was increased by the addition of minor depression cases, thus reflecting an increase in power.

## Results

The over 50’s (aged) study cohort consisted of 643 MZ twin pairs (491 female–female (F–F) and 152 male–male (M–M)) and 577 DZ twin pairs (263 F–F, 73 M–M, 136 female–male (F–M) and 105 male–female (M–F)), with a mean age of 61.30±8.60 (range=50–92). The prevalence of minor depression, major depression and two-category depression was 8.98%, 2.05% and 11.02%, respectively (9.61%, 2.12%, 11.72% in females; 7.38%, 1.88% and 9.26% in males, respectively). Meanwhile, the TE cohort consisted of 1005 MZ twin pairs (609 F–F and 396 M–M) and 1358 DZ twin pairs (455 F–F, 349 M–M, 301 F–M and 253 M–F) with a mean age of 29.80±2.49 (range=23–38). The prevalence of minor depression, major depression and two-category depression was 7.70%, 37.41% and 45.11%, respectively (7.23%, 42.80% and 50.04% in females; 8.32%, 30.33% and 38.65% in males, respectively).

Familial clustering of minor depression and major depression cases was observed (Table 1), with co-twins of minor depression probands having an increased risk of major depression, and *vice versa*, within both cohorts.

No differences in threshold liability distributions were observed within twin pairs, and across sex and zygosity groups, in either study cohort. Importantly, none of the multiple-threshold model goodness-of-fit tests (one for each zygosity group) were significant at the 5% level within the over 50’s study cohort (Table 2). Similarly, only one multiple-threshold model goodness-of-fit test was nominally significant (M–M DZ twin pairs, *P*=0.01) in the TE cohort (Table 2); however, considering goodness-of-fit tests were performed for each of the five zygosity groups and four additional combined groupings, this finding is not considered study-wide significant. Therefore, these results support the validity of the multiple-threshold model for the DSM-IV classifications for minor and major depression, and indicate that they can be conceptualised as different levels of severity on a single dimension of liability.

The polychoric correlations for the varying depression classifications were approximately two times larger in MZ compared to DZ twin pairs within the over 50’s cohort (Table 3). Similarly, with the exception of the MZ non-depressed–minor depression correlation (due to small cell counts), the polychoric correlations for the major depression, two-category depression and three-category depression were at least two to three times higher in MZ compared to DZ twin pairs within the TE cohort (Table 3). The observed MZ>DZ correlations indicate additive genetic factors contribute to the heritability of depression.

The best-fitting model for all depression classifications in the over 50’s cohort was the AE model (Table 4). Similarly, the best-fitting model for major depression, two-category depression and three-category depression was the AE model in the TE cohort.

Within the over 50’s cohort, unique additive genetic factors were estimated to explain ~37% of the heritability of minor depression. Similarly, unique additive genetic factors were estimated to explain ~46% and ~45% of the heritability of major depression, within the over 50’s and TE cohorts, respectively (Table 4). Significantly, the heritability of the two-category model (combining minor depression and major depression) was estimated at 48% (95% CI: 33–62%), which was almost indistinguishable to the three-category model estimate of 47% (95% CI: 33–60%) in the over 50’s cohort. The observed indistinguishability of the estimates for unique additive genetic factors between two-category depression (*A*: 40%, 95% CI: 23–48%) and three-category depression (*A*: 40%, 95% CI: 32–47%) was replicated within the TE cohort. No significant evidence for sex-specific genetic effects was observed within the over 50’s or TE cohorts.

Of the 17 loci robustly associated with major depression in Europeans,^13^ two were nominally (*P*<0.1) associated with major depression in our Australian data set, SNP rs10514299 between *TMEM161B* and *MEF2C* (allele T: odds ratio (OR)=1.10, 95% CI=1.03–1.17; *P*=0.006) and SNP rs11209948 near *NEGR1* (allele T: OR=1.07, 95% CI=1.01–1.12; *P*=0.05). Broadening our case phenotype to include an additional 620 unrelated minor depression cases (providing a total of 4284 unrelated cases) increased the evidence for association with depression at both loci, producing more significant *P*-values of 0.003 and 0.03, respectively. Comparable results were observed utilising a quantitative, three-category depression classification with *P*-values of 0.005 and 0.04, respectively.

## Discussion

Findings from the present study of two independent twin cohorts indicate the heritability of minor depression has a genetic contribution. However, the genetic heritability of minor depression appears larger in the over 50’s cohort compared to the TE cohort. In contrast, the genetic heritability estimates for major depression were comparable at ~46% in individuals over 50 and ~45% in 23–38 year olds. Within each cohort the contribution of additive genetic factors was comparable between the two-category and three-category depression classifications. However, the genetic heritability estimates were larger at 47–48% in the over 50’s cohort compared to 40% in the TE cohort. The differences in heritability estimates between the cohorts are potentially attributable to the time periods assessed within each study cohort (that is, *current* depression in the over 50’s study compared to *lifetime* depression in the TE cohort). In addition, the difference in depression classifications between the study cohorts explains the higher major depression prevalence within the TE cohort. Kendler *et al.*^43^ have reported a comparable elevation in lifetime major depression prevalence within an independent cohort. The authors postulated the higher prevalence of major depression was likely attributable to a lower average cohort age than national population cohorts, and the use of self-report rather than highly structured psychiatric interview—which may underestimate population rates of major depression. In 2016, Zeng *et al.*^44^ showed self-declared depression is a valid alternative to MDD in genetic studies, reporting common genetic effects were highly correlated with significant genetic contributions associated with both classifications.

The near-identical genetic heritability estimates of the two-category and three-category depression classifications and the results of the liability threshold model indicate minor depression and major depression lie on a single genetic-liability continuum, with major depression being more severe but not aetiologically distinct from minor depression. We note that the evidence for a genetic contribution to the heritability of minor depression in younger adults is weak; this is likely due to a relative lack of power (due to the low number of minor depression cases) within the TE cohort. Indeed, the genetic heritability estimate for the broad, two-category depression classification indicates the applicability of a broad depression phenotype is not specific to the over 50’s cohort. Therefore, broadening the depression phenotype in genetic studies by including individuals with a diagnosis of minor depression or major depression should facilitate the identification of genetic risk factors associated with depression due to improved power via increased sample size. Such improved power will be readily provided through re-analysis of existing GWA data sets (which currently exclude minor depression-like cases from analysis), and more cost-effective collection of depression cases in future studies. As previously outlined by Sullivan,^45^ GWA studies will continue to be of great importance for identification of the underlying biology and genetic architecture of psychiatric disorders. Indeed, the MDD working group of the Psychiatric Genomics Consortium has previously emphasised the absence of reference to the underlying biology or pathophysiology within the MDD diagnosis.^11^

Previous MDD GWA analyses have discussed possible approaches to increase power and enable identification of genetic risk loci associated with MDD.^10^ The first approach involves utilising more homogenous MDD case samples. In 2015, the CONVERGE consortium utilised this method by selecting 5303 Han Chinese women with recurrent MDD (of which 85% have the severe melancholic subtype) and 5337 Han Chinese female controls screened to exclude MDD to identify the first SNP loci robustly associated with severe recurring MDD.^12^ Furthermore, in 2017, Power *et al.*^17^ utilised additional phenotypic data to stratify cases and thereby reduce heterogeneity, which enabled the identification of a genetic risk locus associated with MDD onset in adults aged over 27 years. Stratification of MDD cases based on symptom dimensions represents an alternative method of utilising phenotypic data to reduce heterogeneity within GWA studies, with Pearson *et al.*^46^ showing common SNPs explain varying proportions of the variation in the depression symptom dimensions of core depression symptoms, insomnia, appetite and anxiety symptoms (SNP-based heritability=14.3%, 30.3%, 29.6% and 4.7%, respectively). Meanwhile, a complementary approach is to obtain larger sample sizes, which are more representative of the general population. This approach can be achieved by broadly defining depression to detect the common variation of small effect, given the relatively high prevalence and low heritability of MDD.^10^

To demonstrate the utility of using a broader depression phenotype, we examined the association signal of the 17 SNPs reported by Hyde *et al.*,^13^ in Table 2, reaching genome-wide significant association with major depression, utilising our Australian GWA data set. In our Australian sample of 3664 unrelated major depression cases and 7113 unrelated controls, SNP rs10514299 between *TMEM161B* and *MEF2C* and SNP rs11209948 near *NEGR1* were nominally associated with major depression (*P*⩽0.05). Broadening our case phenotype to include an additional 620 unrelated minor depression cases (providing a total of 4284 unrelated cases) increased the statistical evidence for association with depression at both loci. Although a subset (1450 cases and 1711 controls) of the 3664 Australian major depression cases and 7113 controls was part of the Psychiatric Genomics Consortium MDD GWA^11^ that was meta-analysed in Hyde *et al.*,^13^ these results provide proof-of-principle for using a broader depression phenotype to increase power in genetic association studies of depression. In addition, the study by Hyde *et al.*^13^ provides evidence that utilising large self-reported depression data, which broadens the MDD phenotype because of the lack of restriction to clinically validated MDD cases, is an effective strategy for overcoming the large heterogeneity of depression. Further evidence for the utility of broad depression phenotypes in genetic studies is provided by the investigation of depression symptoms conducted by Okbay *et al.*^15^ and MDD or depression symptoms by Direk *et al.*^16^

Continued use of broad depression phenotypes and large cohorts without detailed clinical evaluation, such as from large ongoing commercial (for example, 23 and Me and Kaiser Permanente) and public (for example, UK Biobank and Generation Scotland) data sets, should, therefore, identify additional genetic risk factors and provide the crucial clues to further elucidating the complex molecular pathways underlying MDD—which can then be characterised with respect to particular features of depression via the study of specific patient subgroups in deeply phenotyped clinical cohorts.

## Figures and Tables

**Table 1:** Relative risks^a^ (with their 95% confidence intervals) of depression and fatigue within MZ, DZ_ss_ and DZ_os_ twin pairs

*Proband−co-twin*	*Aged*				*TE*			
	*MZ (643 pairs)*	*DZ*_*ss*_ *(336 pairs)*	*DZ*_*os*_ *F–M (241 pairs)*	*DZ*_*os*_ *M–F (241 pairs)*	*MZ (1005 pairs)*	*DZ*_*ss*_ *(804 pairs)*	*DZ*_*os*_ *F–M (554 pairs)*	*DZ*_*os*_ *M–F (554 pairs)*
MiD−MiD	2.85 (1.62–5.00)	2.67 (1.09–6.51)	1.13 (0.27–4.66)	1.15 (0.29–4.51)	0.91 (0.37–2.23)	1.71 (0.79–3.72)	1.40 (0.49–3.98)	1.05 (0.38-2.85)
MiD−MD	7.32 (2.47–21.67)	2.04 (0.25–16.54)	4.24 (0.82–21.99)	4.02 (0.44–36.69)	1.64 (1.22–2.22)	1.17 (0.82–1.68)	1.06 (0.66–1.70)	1.40 (1.04–1.88)
MiD−MiD/MD	3.48 (2.19–5.54)	2.54 (1.15–5.61)	1.79 (0.66–4.83)	1.51 (0.50–4.53)	1.48 (1.14–1.92)	1.27 (0.95–1.69)	1.12 (0.75–1.66)	1.34 (1.04–1.71)
MD−MiD	5.45 (2.69–11.03)	2.22 (0.35–14.09)	3.53 (0.60–20.66)	3.44 (1.03–11.46)	1.17 (0.75–1.83)	1.06 (0.63–1.80)	1.72 (0.95–3.11)	0.99 (0.53–1.82)
MD−MD	6.02 (0.79–45.56)	—	—	—	2.16 (1.82–2.56)	1.46 (1.22–1.76)	1.16 (0.89–1.50)	1.17 (0.95–1.45)
MD−MiD/MD	5.53 (2.99–10.22)	1.76 (0.28–11.05)	2.79 (0.48–16.07)	3.01 (0.91–9.94)	1.94 (1.68–2.24)	1.39 (1.19–1.63)	1.26 (1.01–1.56)	1.14 (0.95–1.36)
MiD/MD−MiD	3.32 (2.04–5.42)	2.58 (1.12–5.94)	1.46 (0.45–4.75)	1.72 (0.64–4.60)	1.12 (0.73–1.73)	1.17 (0.72–1.91)	1.67 (0.94–2.97)	1.00 (0.57–1.76)
MiD/MD−MD	7.09 (2.50–20.05)	1.64 (0.20–13.42)	3.66 (0.70–19.08)	3.01 (0.33–27.85)	2.07 (1.75–2.45)	1.41 (1.18–1.69)	1.14 (0.89–1.46)	1.22 (1.01–1.48)
MiD/MD−MiD/MD	3.85 (2.54–5.84)	2.39 (1.12–5.06)	1.92 (0.78–4.76)	1.88 (0.79–4.48)	1.86 (1.62–2.14)	1.37 (1.18–1.60)	1.23 (1.00–1.52)	1.18 (1.00–1.39)

Abbreviations: DZ_os_, opposite-sex dizygotic; DZ_ss_, same-sex dizygotic; MD, major depression; MiD, minor depression; MZ, monozygotic; TE, Twin 89 cohort.

a Relative risks and 95% confidence intervals were calculated with respect to non-depressed or non-fatigued status in twin 1. Same-sex twin pair tables were made symmetrical by averaging over using twin 1 or twin 2 as the proband.

**Table 2:** Liability threshold model fit *P*-values

*Twin pair*	*Aged*	*TE*
Complete pairs	0.41	0.53
MZ	0.73	0.75
MZf	0.64	0.81
MZm	0.62	0.92
DZ	0.46	0.43
DZss	0.66	0.66
DZf	0.34	0.21
DZm	0.64	0.01
DZos	0.46	0.46

Abbreviations: DZ, dizygotic; DZf, DZ female; DZm, DZ male; DZos, DZ opposite sex; DZss, DZ same sex; MZ, monozygotic; MZf, MZ female; MZm, MZ male; TE, Twin 89 cohort.

Aged: over 50’s (aged) cohort.

**Table 3:** Polychoric correlations with their 95% confidence intervals for depression according to zygosity

*Depression classification*	*Aged*		*TE*	
	*MZ*	*DZ*	*MZ*	*DZ*
Non-depressed, minor depression	0.37 (0.17–0.56)	0.21 (−0.04–0.45)	0.05 (−0.22–0.31)	0.17 (−0.04–0.39)
Non-depressed, major depression	0.46 (−0.01–0.93)	—	0.49 (0.41–0.58)	0.19 (0.10–0.28)
Non-depressed, minor or major depression	0.49 (0.33–0.64)	0.25 (0.05–0.46)	0.43 (0.34–0.51)	0.18 (0.10–0.27)
Non-depressed, minor depression, major depression	0.48 (0.33–0.62)	0.24 (0.04–0.43)	0.43 (0.35–0.51)	0.17 (0.09–0.25)

Abbreviations: DZ, dizygotic; MZ, monozygotic; TE, Twin 89 cohort.

Aged: over 50’s (aged) cohort.

**Table 4:** Fit statistics and variance estimates (with their 95% confidence intervals) from univariate structural equation modelling

*Model*	*Aged*										*TE*			
	*−2LL*	P*-value (ACE)*	P*-value (ADE)*	*AIC*	*A*	*C (or D)*	*E*	*−2LL*	P*-value (ACE)*	P*-value (ADE)*	*AIC*	*A*	*C (or D)*	*E*
*Non-depressed, minor depression*														
ACE	1446.58	—	—	−3323.42	0.32 (0.00–0.54)	0.05 (0.00–0.43)	0.63 (0.46–0.84)	2203.82	—	—	−3702.18	0.00 (0.00–0.35)	0.14 (0.00–0.30)	0.87 (0.65–1.00)
AE	1446.61	0.87	1.00	−3325.40	0.37 (0.18–0.54)	—	0.63 (0.46–0.82)	2204.58	0.39	1.00	−3703.43	0.15 (0.00–0.37)	—	0.85 (0.63–1.00)
CE	1447.58	0.32	—	−3324.42	—	0.30 (0.13–0.45)	0.70 (0.46–0.82)	2203.82	1.00	—	−3704.18	—	0.14 (0.00–0.30)	0.87 (0.70–1.00)
E	1460.62	8.9 × 10^−4^	9.0 × 10^−4^	−3313.38	—	—	1.00 (1.00–1.00)	2206.15	0.31	0.46	−3703.85	—	—	1.00 (1.00–1.00)
ADE	1446.61	—	—	−3323.40	0.37 (0.00–0.54)	0.00 (0.00–0.54)	0.63 (0.45–0.82)	2204.58	—	—	−3701.43	0.15 (0.00–0.37)	0.00 (0.00–0.36)	0.85 (0.63–1.00)
														
*Non-depressed, major depression*														
ACE	475.73	—	—	−3956.27	0.46 (0.00–0.83)	0.00 (0.00–0.62)	0.54 (0.17–1.00)	5717.52	—	—	−2996.48	0.45 (0.29–0.53)	0.00 (0.00–0.12)	0.55 (0.47–0.63)
AE	475.73	1.00	0.49	−3958.27	0.46 (0.00–0.83)	—	0.54 (0.17–1.00)	5717.517	1.00	0.28	−2998.48	0.45 (0.37–0.53)	—	0.55 (0.47–0.63)
CE	476.73	0.32	—	−3957.27	—	0.29 (0.00–0.66)	0.71 (0.34–1.00)	5735.63	2.1 × 10^−5^	—	−2980.37	—	0.31 (0.24–0.38)	0.69 (0.62–0.76)
E	478.03	0.32	0.25	−3957.97	—	—	1.00 (1.00–1.00)	5813.19	1.7 × 10^−21^	9.4 × 10^−22^	−2904.81	—	—	1.00 (1.00–1.00)
ADE	475.27	—	—	−3956.74	0.00 (0.00–0.81)	0.51 (0.00–0.85)	0.49 (0.15–1.00)	5716.37	—	—	−2997.64	0.25 (0.00–0.52)	0.23 (0.00–0.55)	0.52 (0.44–0.62)
														
*Two-category depression (non-depressed, minor depression or major depression)*														
ACE	1655.41	—	—	−3214.59	0.44 (0.00–0.61)	0.04 (0.00–0.45)	0.52 (0.38–0.69)	6353.91	—	—	−3088.09	0.40 (0.22–0.48)	0.00 (0.00–0.14)	0.60 (0.52–0.68)
AE	1655.44	0.88	1.00	−3216.56	0.48 (0.33–0.62)	0.52 (0.38–0.67)	6353.91	1.00	0.56	−3090.09	0.40 (0.32–0.48)	—	0.50 (0.52–0.68)	
CE	1658.31	0.09	—	−3213.69	—	0.51 (0.39–0.49)	0.61 (0.49–0.74)	6368.07	2.0 × 10^−4^	—	−3075.93	—	0.28 (0.22–0.34)	0.72 (0.66–0.78)
E	1690.12	2.9 × 10^−8^	2.9 × 10^−8^	−3183.88	—	—	1.00 (1.00–1.00)	6445.22	1.5 × 10^−20^	1.3 × 10^−20^	−3000.78	—	—	1.00 (1.00–1.00)
ADE	1655.44	—	—	−3214.56	0.48 (0.00–0.62)	0.00 (0.00–0.60)	0.52 (0.38–0.67)	6353.57	—	—	−3088.43	0.31 (0.00–0.48)	0.11 (0.00–0.47)	0.58 (0.50–0.67)
														
*Three-category depression (non-depressed, minor depression, major depression)*														
ACE	1912.56	—	—	−2955.44	0.47 (0.00–0.60)	0.01 (0.00–0.41)	0.53 (0.40–0.69)	8281.43	—	—	−1158.57	0.40 (0.24–0.47)	0.00 (0.00–0.00)	0.60 (0.53–0.68)
AE	1912.56	0.99	1.00	−2957.44	0.47 (0.32–0.60)	—	0.53 (0.40–0.67)	8281.43	1.00	0.42	−1160.57	0.40 (0.32–0.47)	—	0.60 (0.53–0.68)
CE	1916.14	0.06	—	−2953.86	—	0.37 (0.25–0.49)	0.53 (0.51–0.75)	8298.23	4.2 × 10^−5^	—	−1143.78	—	0.28 (0.22–0.33)	0.72 (0.67–0.78)
E	1948.85	1.3 × 10^−8^	1.3 × 10^−8^	−2923.15	—	—	1.00 (1.00–1.00)	8381.43	1.9 × 10^−22^	1.4 × 10^−22^	−1062.57	—	—	1.00 (1.00–1.00)
ADE	1912.56	—	—	−2955.44	0.47 (0.00–0.60)	0.01 (0.00–0.59)	0.53 (0.40–0.67)	8280.79	—	—	−1159.21	0.27 (0.00–0.46)	0.14 (0.00–0.14)	0.59 (0.51–0.67)

Abbreviations: A, additive genetic component; AIC, Akaike’s Information Criteria; C, common environmental component; E, unique environmental component; TE, Twin 89 cohort; −2LL, minus two log likelihood.

Aged: over 50’s (aged) cohort.

## References

[bib1] Sullivan PF, Neale MC, Kendler KS. Genetic epidemiology of major depression: review and meta-analysis. Am J Psychiatry 2000; 157: 1552–1562. 11007705 10.1176/appi.ajp.157.10.1552

[bib2] Sullivan PF, de Geus EJ, Willemsen G, James MR, Smit JH, Zandbelt T et al. Genome-wide association for major depressive disorder: a possible role for the presynaptic protein piccolo. Mol Psychiatry 2009; 14: 359–375. 19065144 10.1038/mp.2008.125 PMC2717726

[bib3] Muglia P, Tozzi F, Galwey NW, Francks C, Upmanyu R, Kong XQ et al. Genome-wide association study of recurrent major depressive disorder in two European case-control cohorts. Mol Psychiatry 2010; 15: 589–601. 19107115 10.1038/mp.2008.131

[bib4] Lewis CM, Ng MY, Butler AW, Cohen-Woods S, Uher R, Pirlo K et al. Genome-wide association study of major recurrent depression in the U.K. population. Am J Psychiatry 2010; 167: 949–957. 20516156 10.1176/appi.ajp.2010.09091380

[bib5] Rietschel M, Mattheisen M, Frank J, Treutlein J, Degenhardt F, Breuer R et al. Genome-wide association-, replication-, and neuroimaging study implicates HOMER1 in the etiology of major depression. Biol Psychiatry 2010; 68: 578–585. 20673876 10.1016/j.biopsych.2010.05.038

[bib6] Shi J, Potash JB, Knowles JA, Weissman MM, Coryell W, Scheftner WA et al. Genome-wide association study of recurrent early-onset major depressive disorder. Mol Psychiatry 2011; 16: 193–201. 20125088 10.1038/mp.2009.124 PMC6486400

[bib7] Shyn SI, Shi J, Kraft JB, Potash JB, Knowles JA, Weissman MM et al. Novel loci for major depression identified by genome-wide association study of sequenced treatment alternatives to relieve depression and meta-analysis of three studies. Mol Psychiatry 2011; 16: 202–215. 20038947 10.1038/mp.2009.125 PMC2888856

[bib8] Kohli MA, Lucae S, Saemann PG, Schmidt MV, Demirkan A, Hek K et al. The neuronal transporter gene SLC6A15 confers risk to major depression. Neuron 2011; 70: 252–265. 21521612 10.1016/j.neuron.2011.04.005 PMC3112053

[bib9] Bosker FJ, Hartman CA, Nolte IM, Prins BP, Terpstra P, Posthuma D et al. Poor replication of candidate genes for major depressive disorder using genome-wide association data. Mol Psychiatry 2011; 16: 516–532. 20351714 10.1038/mp.2010.38

[bib10] Wray NR, Pergadia ML, Blackwood DH, Penninx BW, Gordon SD, Nyholt DR et al. Genome-wide association study of major depressive disorder: new results, meta-analysis, and lessons learned. Mol Psychiatry 2012; 17: 36–48. 21042317 10.1038/mp.2010.109 PMC3252611

[bib11] Major Depressive Disorder Working Group of the Psychiatric GWAS ConsortiumMajor Depressive Disorder Working Group of the Psychiatric GWAS ConsortiumRipke S Major Depressive Disorder Working Group of the Psychiatric GWAS ConsortiumWray NR Major Depressive Disorder Working Group of the Psychiatric GWAS ConsortiumLewis CM Major Depressive Disorder Working Group of the Psychiatric GWAS ConsortiumHamilton SP Major Depressive Disorder Working Group of the Psychiatric GWAS ConsortiumWeissman MM et al. A mega-analysis of genome-wide association studies for major depressive disorder. Mol Psychiatry 2013; 18: 497–511. 22472876 10.1038/mp.2012.21 PMC3837431

[bib12] CONVERGE consortium. Sparse whole-genome sequencing identifies two loci for major depressive disorder. Nature 2015; 523: 588–591. 26176920 10.1038/nature14659 PMC4522619

[bib13] Hyde CL, Nagle MW, Tian C, Chen X, Paciga SA, Wendland JR et al. Identification of 15 genetic loci associated with risk of major depression in individuals of European descent. Nat Genet 2016; 48: 1031–1036. 27479909 10.1038/ng.3623 PMC5706769

[bib14] Visscher PM, Brown MA, McCarthy MI, Yang J. Five years of GWAS discovery. Am J Hum Genet 2012; 90: 7–24. 22243964 10.1016/j.ajhg.2011.11.029 PMC3257326

[bib15] Okbay A, Baselmans BML, De Neve J-E, Turley P, Nivard MG, Fontana MA et al. Genetic variants associated with subjective well-being, depressive symptoms, and neuroticism identified through genome-wide analyses. Nat Genet 2016; 48: 624–633. 27089181 10.1038/ng.3552 PMC4884152

[bib16] Direk N, Williams S, Smith JA, Ripke S, Air T, Amare AT et al. An analysis of two genome-wide association meta-analyses identifies a new locus for broad depression phenotype. Biol Psychiatry 2016. 10.1016/j.biopsych.2016.11.013 PMC5462867 28049566

[bib17] Power RA, Tansey KE, Buttenschon HN, Cohen-Woods S, Bigdeli T, Hall LS et al. Genome-wide association for major depression through age at onset stratification: Major Depressive Disorder Working Group of the Psychiatric Genomics Consortium. Biol Psychiatry 2017; 81: 325–335. 27519822 10.1016/j.biopsych.2016.05.010 PMC5262436

[bib18] American Psychiatric AssociationDiagnostic and Statistical Manual of Mental Disorders, Fifth Edition (DSM-V). American Psychiatric Publishing:: Washington, DC, USA, 2013.

[bib19] American Psychiatric Association.Diagnostic and Statistical Manual of Mental Disorders DSM-IV-TR. American Psychiatric Association:: Washington, DC, USA, 2000.

[bib20] Cuijpers P, Smit F. Subthreshold depression as a risk indicator for major depressive disorder: a systematic review of prospective studies. Acta Psychiatr Scand 2004; 109: 325–331. 15049768 10.1111/j.1600-0447.2004.00301.x

[bib21] Judd LL, Akiskal HS, Paulus MP. The role and clinical significance of subsyndromal depressive symptoms (SSD) in unipolar major depressive disorder. J Affect Disord 1997; 45: 5–18. 9268771 10.1016/s0165-0327(97)00055-4

[bib22] Chen LS, Eaton WW, Gallo JJ, Nestadt G, Crum RM. Empirical examination of current depression categories in a population-based study: Symptoms, course, and risk factors. Am J Psychiatry 2000; 157: 573–580. 10739416 10.1176/appi.ajp.157.4.573

[bib23] Rapaport MH, Judd LL, Schettler PJ, Yonkers KA, Thase ME, Kupfer DJ et al. A descriptive analysis of minor depression. Am J Psychiatry 2002; 159: 637–643. 11925303 10.1176/appi.ajp.159.4.637

[bib24] Lewinsohn PM, Klein DN, Durbin EC, Seeley JR, Rohde P. Family study of subthreshold depressive symptoms: Risk factor for MDD? J Affect Disord 2003; 77: 149–157. 14607392 10.1016/s0165-0327(02)00106-4

[bib25] Cuijpers P, de Graaf R, van Dorsselaer S. Minor depression: risk profiles, functional disability, health care use and risk of developing major depression. J Affect Disord 2004; 79: 71–79. 15023482 10.1016/S0165-0327(02)00348-8

[bib26] Bucholz KK, Heath AC, Madden PAF, Slutske WS, Statham DJ, Dunne MP et al. Drinking in an older population: cross-sectional and longitudinal data from the Australian twin registry. In: Gomberg EL, Hegedus AM, Zucker RA (eds). Alcohol Problems and Aging. National Institutes of Health: Bethesda, MD, USA, 1998, pp 41–62..

[bib27] Mosing MA, Medland SE, McRae A, Landers JG, Wright MJ, Martin NG. Genetic influences on life span and its relationship to personality: a 16-year follow-up study of a sample of aging twins. Psychosom Med 2012; 74: 16–22. 22155943 10.1097/PSY.0b013e3182385784

[bib28] Goldberg DP, Blackwell B. Psychiatric illness in general practice. A detailed study using a new method of case identification. Br Med J 1970; 1: 439–443. 5420206 10.1136/bmj.2.5707.439 PMC1700485

[bib29] Bedford A, Deary IJ. The personal disturbance scale (DSSI/sAD): development, use and structure. Pers Individ Dif 1997; 22: 493–510.

[bib30] Corfield EC, Martin NG, Nyholt DR. Co-occurrence and symptomatology of fatigue and depression. Compr Psychiatry 2016; 71: 1–10. 27567301 10.1016/j.comppsych.2016.08.004

[bib31] Heath AC, Howells W, Kirk KM, Madden PA, Bucholz KK, Nelson EC et al. Predictors of non-response to a questionnaire survey of a volunteer twin panel: findings from the Australian 1989 twin cohort. Twin Res 2001; 4: 73–80. 11665339 10.1375/1369052012182

[bib32] Yang Y, Zhao H, Heath AC, Madden PA, Martin NG, Nyholt DR. Shared genetic factors underlie migraine and depression. Twin Res Hum Genet 2016; 19: 341–350. 27302564 10.1017/thg.2016.46 PMC5436908

[bib33] Nyholt DR, Gillespie NG, Heath AC, Merikangas KR, Duffy DL, Martin NG. Latent class and genetic analysis does not support migraine with aura and migraine without aura as separate entities. Genet Epidemiol 2004; 26: 231–244. 15022209 10.1002/gepi.10311

[bib34] Reich T, James JW, Morris CA. The use of multiple thresholds in determining the mode of transmission of semi-continuous traits. Ann Hum Genet 1972; 36: 163–184. 4676360 10.1111/j.1469-1809.1972.tb00767.x

[bib35] Uebersax JS. User Guide for POLYCORR 1.1. (Statistical Methods for Rater Agreement web site). Available from: http://john-uebersax.com/stat/xpc.htm (accessed on 2007).

[bib36] Kendler KS. Twin studies of psychiatric illness. Current status and future directions. Arch Gen Psychiatry 1993; 50: 905–915. 8215816 10.1001/archpsyc.1993.01820230075007

[bib37] Neale M, Cardon L. Methodology for Genetic Studies of Twins and Families. Springer: New York, NY, 1992.

[bib38] Boker S, Neale M, Maes H, Wilde M, Spiegel M, Brick T et al. OpenMx: an open source extended structural equation modeling framework. Psychometrika 2011; 76: 306–317. 23258944 10.1007/s11336-010-9200-6 PMC3525063

[bib39] Akaike H. A new look at the statistical model identification. IEEE Trans Automatic Control 1974; 19: 716–723.

[bib40] Akaike H. Information theory and an extension of the maximum likelihood principle. In: Petrov BN, Csáki F (eds). Second International Symposium on Information Theory; Tsahkadsor, Armenia, USSR. Akadémiai Kiadó: Budapest, 1973, pp 267–281..

[bib41] Medland SE, Nyholt DR, Painter JN, McEvoy BP, McRae AF, Zhu G et al. Common variants in the trichohyalin gene are associated with straight hair in Europeans. Am J Hum Genet 2009; 85: 750–755. 19896111 10.1016/j.ajhg.2009.10.009 PMC2775823

[bib42] Purcell S, Neale B, Todd-Brown K, Thomas L, Ferreira MAR, Bender D et al. PLINK: a tool set for whole-genome association and population-based linkage analyses. Am J Hum Genet 2007; 81: 559–575. 17701901 10.1086/519795 PMC1950838

[bib43] Kendler KS, Neale MC, Kessler RC, Heath AC, Eaves LJ. A population-based twin study of major depression in women. The impact of varying definitions of illness. Arch Gen Psychiatry 1992; 49: 257–266. 1558459 10.1001/archpsyc.1992.01820040009001

[bib44] Zeng Y, Navarro P, Xia C, Amador C, Fernandez-Pujals AM, Thomson PA et al. Shared genetics and couple-associated environment are major contributors to the risk of both clinical and self-declared depression. EBioMedicine 2016; 14: 161–167. 27838479 10.1016/j.ebiom.2016.11.003 PMC5161419

[bib45] Sullivan P. Don't give up on GWAS. Mol Psychiatry 2012; 17: 2–3. 21826059 10.1038/mp.2011.94 PMC4187109

[bib46] Pearson R, Palmer RH, Brick LA, McGeary JE, Knopik VS, Beevers CG. Additive genetic contribution to symptom dimensions in major depressive disorder. J Abnorm Psychol 2016; 125: 495–501. 27124715 10.1037/abn0000161 PMC4852390

